# Coping During the COVID-19 Pandemic: A Qualitative Study of Older Adults Across the United States

**DOI:** 10.3389/fpubh.2021.643807

**Published:** 2021-04-07

**Authors:** Jessica M. Finlay, Jasdeep S. Kler, Brendan Q. O'Shea, Marisa R. Eastman, Yamani R. Vinson, Lindsay C. Kobayashi

**Affiliations:** ^1^Social Environment and Health Program, Survey Research Center, Institute for Social Research, University of Michigan, Ann Arbor, MI, United States; ^2^Department of Epidemiology, Center for Social Epidemiology and Population Health, University of Michigan School of Public Health, Ann Arbor, MI, United States; ^3^Department of Health Management and Policy, University of Michigan School of Public Health, Ann Arbor, MI, United States

**Keywords:** aging, mental health, resilience, coping strategies, qualitative methods

## Abstract

**Objective:** Older adults may struggle with stresses and daily life challenges associated with the Coronavirus Disease 2019 (COVID-19) pandemic. Yet they may also utilize emotional and behavioral coping strategies. This qualitative paper aims to identify ways of coping with worries and stress during the pandemic from the perspectives of older adults in the United States.

**Methods:** The COVID-19 Coping Study recruited 6,938 adults aged ≥55 through online multi-frame sampling from April 2-May 31, 2020 across all 50 US states, the District of Columbia, and Puerto Rico. The online questionnaire focused on the effects of COVID-19 on daily life, mental health, and well-being. This included an open-ended question regarding participants' coping strategies. We used qualitative content analysis to identify and code diverse coping strategies. Our general inductive approach enabled findings to emerge from the most frequent and dominant themes in the raw data.

**Results:** A total of 5,180 adults [74% of the total sample; mean age 67.3 (SD 7.9); 63.8% female] responded to the question about using strategies to cope with living through the COVID-19 pandemic. Frequently-reported strategies included exercising and going outdoors, modifying routines, following public health guidelines, adjusting attitudes, and staying socially connected. Some coping strategies were health-limiting (e.g., overeating), while most strategies encouraged self-improvement, positive adjustment, and wellness.

**Conclusions:** This study provides novel qualitative evidence on coping strategies of older adults early in the COVID-19 pandemic. Findings can inform community and clinical interventions to support older adults that harness positive coping strategies such as exercise, modified routines, and social strategies to improve physical and mental health, foster social support, and encourage meaningful daily activities during times of stress and trauma.

## Introduction

The Coronavirus Disease 2019 (COVID-19) pandemic has dire and immediate consequences for the health and well-being of aging populations. Older adults, especially those with comorbid health conditions, are at elevated risk of COVID-19 morbidity and mortality compared to younger population groups (1–3). Beyond physical illness, the pandemic exposes older people to myriad life challenges including disrupted plans, frustration and boredom, separation from family and friends, irregular access to supplies (e.g., food, medication), and financial strain (4, 5). Older adults may also be at heightened risk of pandemic-related personal losses such as bereavement of a family member or friend, job loss or reduced income if not retired, and long-term exclusion from participation in social and public activities (6).

Public health strategies to limit transmission of SARS-CoV-2, the virus that causes COVID-19, have included stay-at-home orders, physical distancing guidelines, group gathering restrictions, cancellation of planned social and public events, and travel restrictions. These interventions, which are necessary in the absence of widespread vaccinations, can cut off social support networks, restrict access to services, and make people feel anxious and unsafe (5). Older adults who were socially isolated and lonely pre-pandemic (7) may be at heightened risk for emotional distress and poor mental health (8). Media coverage of widespread hospitalizations and deaths among older adults during the pandemic, in addition to ageism in public discourse, can portray older adults as helpless, frail, and burdensome on society (9, 10). This may have lasting physical and mental health impacts (8, 11).

Given the immense burden of COVID-19 on aging populations, it is essential to understand effective ways of coping with living through the pandemic. This paper contributes to an emerging counternarrative in response to ageist portrayals of older adults in popular discourse as vulnerable, frail, and disposable in the context of the COVID-19 pandemic [see also (4, 9)]. We shift from this negative perspective to focus on the psychosocial strengths and resiliencies of older adults. Resilience has many definitions and is difficult to measure. Previous research suggests that older adults with high psychological resilience are better able to utilize internalized recourses that may help buffer the negative effects of experiencing adversity (12, 13). Coping involves cognitive and behavioral strategies that individuals employ to deal with or control stressful circumstances, and can be impacted by multiple biological and psychosocial factors including physical health, personality, spirituality, and social support. Active coping involves behaviors to proactively address, modify, or overcome a stressor or situation. Regulatory coping refers to reflection about the stressor in order to reduce its effects, such as reframing a stressor or adapting through a change in attitude, expectation, or perception. An individual can consciously or subconsciously employ both types of strategies concurrently (6, 14). Not all coping strategies are successful or helpful. Denial, for example, may not be the most appropriate response to a problem, but is a frequently-used cognitive coping strategy as people age (15).

This qualitative study aims to fill an evidence gap on the coping strategies employed by older adults since the pandemic onset. Quantitative epidemiological models can miss important social implications of the disease and public health strategies (16). Qualitative insights are needed to generate novel insights and more comprehensive understanding of complex realities, nuanced lived experiences, and how people are making sense of and dealing with what is happening around them during this collective trauma. This paper contributes new knowledge of multiple subjective realities, viewpoints, meanings, and motivations among aging adults since the pandemic onset. Over 5,000 Americans aged 55 years and over in the COVID-19 Coping Study shared their personal strategies for coping during the early months of the pandemic. The qualitative results of our study show profound psychosocial resiliency among these older adults by highlighting specific strategies used to cope with adversities of the pandemic.

## Research Design and Methods

### Data Collection

This manuscript analyzes open-ended responses to the question: “Are you using any strategies that have been helping you to cope with the COVID-19 (coronavirus) pandemic? Please describe them.” This question was part of the COVID-19 Coping Study, a longitudinal, mixed-methods study of adults aged 55 and older residing in the US [see detailed methodology in (17)]. The overall study aims to investigate how social, behavioral, and economic impacts of the COVID-19 pandemic affect the mental health and well-being of older adults.

We used a multi-frame online recruitment strategy from April 2 to May 31, 2020, to enhance coverage of diverse populations and geographic locations. This included a snowball sample recruited through social media, organization mailing lists, volunteer databases, and word-of-mouth. A panel sample was recruited from an existing online research panel maintained by a professional survey company. Quotas for age, gender, race, ethnicity, and education matched the US population aged 55 and older. The snowball sample participants did not receive compensation, while panel sample participants received a nominal amount.

The online baseline questionnaire (available in English and Spanish) assessed sociodemographic factors; employment and living situations; COVID-19 testing and symptom history for self, family, and friends; physical and mental health; physical distancing practices; changes to daily life during COVID-19; sources of worry and stress; and ways of coping with stress during the pandemic. Data for this analysis was collected April 2-May 31, 2020. Monthly follow-up surveys are ongoing (May 2020–May 2021), and will transition to annual follow-up surveys in May 2022. The University of Michigan Health Sciences and Behavioral Sciences Institutional Review Board approved the study protocol (HUM00179632), and all participants provided written informed consent.

### Analysis

We used qualitative content analysis to interpret the text data through a systematic classification process of coding followed by identifying themes and patterns (18). This method supported immersion in the data to enable new insights to emerge and inductively develop categories without imposing preconceived categories (19). Content analysis enables words to be distilled into fewer content-related categories. When classified into the same categories, words and phrases are assumed to share the same meaning (20). A challenge of content analysis is its flexibility and that there is no simple or “right” way of doing it. Each inquiry is distinctive (21), and different researchers are likely to produce non-identical findings (22). An advantage of the method is that large volumes of textual data can be incorporated into the analysis (20), which was necessary given our extremely large sample size and hundreds of pages of open-ended responses. While initially daunting and overall a time-consuming process, we systematically worked through the data with our research question in mind: *What strategies are participants utilizing to help cope with the pandemic?* Our team-based approach and focus on a single question enabled immersion in the data to develop our findings (23).

We followed a general inductive approach (22, 24, 25) to enable our findings to emerge from the most frequent and dominant themes in the raw data, without the restraints imposed by more structured methodologies such as those used in deductive experimental and hypothesis testing research. This is consistent with Strauss and Corbin's (26) description: “The researcher begins with an area of study and allows the theory to emerge from the data” (p. 12). While this approach is not as strong as some other analytic strategies for theory or model development (22), its straightforward and systematic approach suited our research purposes and quality and quantity of data.

We developed categories from the raw data into a framework that captured key themes and processes through multiple interpretations. The process involved five steps: First, immersion, in which the first three authors (JMF, JSK, BQO) independently read all data repeatedly to gain a sense of the whole. While an extremely large sample size, this analysis focused on just one open-ended question (as opposed to analyzing multi-question interviews or focus groups, for example). Participants responses on coping strategies ranged from 1-word to multi-paragraph, with the majority succinct in length (i.e., short phrases to two-sentence responses). This kept the textual volume manageable for each author to read and review. Each coder wrote notes and headings while reading the data to develop potential codes, and re-read the material to ensure that as many headings as necessary were written down in the margins to describe all aspects of the content (20). Second, all authors developed the coding scheme: an inductive approach in which codes flowed directly from the data. We discussed and defined the coding scheme through procedural rules. The first three authors test coded material in Excel and NVivo 12 to check for consistency in coding. Third, JSK read all data to assign words and phrases into relevant codes in Excel. JSK added additional codes, if necessary, after discussion and deliberation with co-authors. JMF and BQO reviewed the coding for consistency and completeness. Fourth, all authors reviewed material to share thoughts, impressions, and major takeaways. We sorted codes into categories based upon how different codes were meaningfully related and linked to each other. Fifth, all authors prepared to report findings as a framework by finalizing the names and definitions of each category and code, counting frequencies of categories and codes, summarizing themes, and selecting exemplar quotes. We enhanced methodological rigor through multiple strategies: (1) peer debriefing where we met with non-study team members to review and discuss our methods, emerging findings, and potential researcher biases; (2) referential adequacy in which the first three authors coded and archived the snowball sample responses, and then coded the panel sample to test the validity of findings; (3) negative case analysis where we modified and refined our conclusions by searching for and incorporating contradictory cases; (4) member checking by sending preliminary findings to study participants in a newsletter to invite feedback and critical discussion; and (5) clear audit trails to transparently describe the steps taken from initial data collection to development and reporting of findings (27).

## Results

Of the 6,938 total participants in the COVID-19 Coping Study, 5,180 (74.6%) wrote a response to the open-ended coping strategies question (Table 1). These respondents were nearly two-thirds female, on average 67 years old (SD = 7.9), and largely White (87.2%). Eighty percent of responses were from participants with at least some college education, nearly two-thirds were married or in a relationship, one-quarter lived alone, and half were retired (Table 1).

**Table 1:** Baseline characteristics of the sample, COVID-19 Coping Study, 2020.

**Characteristic**	***N***	**(%)**
**Total N**	**5,180**	**100.0**
**Sex/gender (*****N*** **= 5,177)**		
Male	1,870	(36.1)
Female	3,302	(63.8)
Other	5	(0.1)
Age; mean (SD)	67.3	(7.9)
**Race**		
White	4,518	(87.2)
Black	335	(6.5)
Asian	160	(3.1)
American Indian or Alaska Native	29	(0.6)
Native Hawaiian or Other Pacific Islander	1	(<0.1)
Other Race	53	(1.0)
Two or more races	84	(1.6)
**Ethnicity (*****N*** **= 5,108)**		
Hispanic or Latinx	283	(5.5)
**Highest level of education**		
Education: Less than high school	139	(2.7)
Education: High school diploma or equivalency	876	(16.9)
Education: Some college or 2-year associate degree	1,017	(19.6)
Education: 4-year college or University degree	1,350	(26.1)
Education: Postgraduate or professional degree	1,798	(34.7)
**Relationship status (*****N*** **= 5,167)**		
Single, never married	429	(8.3)
Single, divorced/separated	873	(16.9)
Single, widowed	514	(9.9)
Married or in a relationship	3351	(64.9)
**Living arrangement (*****N*** **= 5,137)**		
Living Alone	1,378	(26.8)
**Employment status pre-COVID-19 (5,178)**		
Employed	1,972	(38.1)
Unemployed	515	(9.9)
Retired	2,691	(52.0)

The analysis generated 14 categories of coping strategies (Table 2). On average, participants reported coping strategies related to 1.6 categories of strategies (SD = 0.95). The most common coping categories were related to exercise and the outdoors (reported by 26% of respondents, 1,349/5,180), daily life (25%, 1,294/5,180), COVID-19 precautions (18.9%, 980/5,180), attitude and outlook (16.1%, 835/5,180), and social connections (15.3%, 792/5,180). Over twenty percent of respondents (1,112/5,180) explicitly reported *not* using any coping strategies. Below we describe each strategy category, in order of frequency, with illustrative quotes from participants.

**Table 2:** Descriptions and prevalence of coping strategies in the sample, COVID-19 Coping Study, 2020 (*n* = 5,180).

**Categories**	**Description of sub-categories**	**Frequency**	**Percent of responses (%)**
**Attitude and outlook**		**835**	**16.1**
Laughter and humor	Seeking out and enjoying laughter and humor	49	0.9
Meditation and breathing	Practicing meditation, mindfulness, and breathing exercises	339	6.5
Relaxing and staying calm	Trying to be relaxed and stay calm	53	1.0
Staying positive and being grateful	Staying positive, being grateful, reframing perspective to positive, accepting the situation	394	7.6
**COVID-19 precautions**		**980**	**18.9**
Cleaning	Sanitary cleaning: spraying surfaces, disinfecting, wiping down objects	68	1.3
Following public health guidelines	Following COVID-19 guidelines to reduce exposure	912	17.6
**Civic engagement**		**211**	**4.1**
Helping others and donating	Helping others, donating money, making masks to donate, supporting local businesses	187	3.6
Political engagement and activism	Political engagement, activism, volunteering on campaigns, contacting elected officials	24	0.5
**Daily life**		**1,294**	**25.0**
Cooking or baking	Cooking or baking food	94	1.8
Got a pet or are taking care of animal	Getting a new pet or taking care of animal(s)	27	0.5
Hobbies	Engaging in hobbies (e.g., writing, arts and crafts, woodworking, puzzles, crochet and knitting, games)	406	7.8
Keeping a routine	Making to-do lists and sticking to a routine (either new, modified, or well-honed)	234	4.5
Listening to audio content	Listening to music, podcasts, radio shows, or audiobooks	70	1.4
Reading	Reading books	214	4.1
Specified keeping busy or occupied	Expressed efforts to keep busy or occupied	177	3.4
Working	Working more, focusing on work, or earning more money	72	1.4
**Exercise and the outdoors**		**1,349**	**26.0**
Exercise	Exercising (e.g., walking, yoga, biking, golfing, jogging, swimming)	883	17.0
Outdoor activities	Outdoor activities of any type (includes walking dog and going for a drive)	466	9.0
**Faith-based practices**		**363**	**7.0**
Religious practices	Religious activity (e.g., prayer, reading scripture, watching or participating in faith services)	363	7.0
**Food, alcohol, and substances**		**190**	**3.7**
Cannabis/marijuana use	Using cannabis/marijuana	15	0.3
Drinking alcohol	Drinking alcohol (e.g., beer, wine, spirits)	19	0.4
Eating more	Eating more food	7	0.1
Ordering food or takeout	Ordering food or takeout meals	14	0.3
Trying to consume responsibly	Try to eat healthier, eat good food, take vitamins, stay hydrated, drink tea, drink less alcohol	135	2.6
**Health and wellness**		**158**	**3.1**
Getting more or better sleep/rest	Sticking to a sleep schedule, or getting more or better-quality sleep and rest	62	1.2
Seeking health support	Seeking physical and/or mental health professional/s, or support group	63	1.2
Taking medication	Taking medication (CBD, medication, sleep aid, light therapy)	33	0.6
**Nothing**		**1,112**	**21.5**
None	Not being able to cope or not having to cope	1,112	21.5
**Online and media engagement**		**577**	**11.1**
Limiting news intake or social media	Limiting news intake or social media	253	4.9
Online activities or computer time	Spending time on the computer or with online activities	38	0.7
Shopping online	Shopping online	2	0.0
Watching or reading the news to stay informed	Watching or reading the news, or staying informed	165	3.2
Watching video content	Watching movies and TV shows (not the news)	119	2.3
**Other**		**115**	**2.2**
Don't Understand	Do not understand the question	19	0.4
Other	Other (does not fit any categories)	96	1.9
**Planning**		**44**	**0.8**
Planning for the present or future	Planning for the present and future, care directives, estate planning, vacations	44	0.8
**Projects and learning**		**265**	**5.1**
Home projects	Doing home projects (e.g., building things, organizing, big spring cleaning)	143	2.8
Learning something new or taking a course	Learning something new, or taking an educational course	82	1.6
Projects (unspecified)	Projects that don't fall in the other categories (unspecified projects, programming projects)	40	0.8
**Social connection**		**792**	**15.3**
Contacting others	Contacting/reaching out to and communicating with family, friends, spouse, neighbors, and others by phone, text message, or video call	613	11.8
Spending in-person time with others	Spending time in-person with family, friends, spouse, or neighbors; watching grandchildren, or other in-person caregiving	140	2.7
Support groups and recovery programs	AA meetings, 12 Steps program, or other recovery program	16	0.3
Using social media or chat rooms	Posting on social media, or joining chatrooms	23	0.4

### Exercise and the Outdoors

Exercise, particularly going for walks and doing yoga, were frequently described coping strategies to improve physical and mental well-being. Cheryl (57y, F)^1^ shared: “Walking outdoors morning and afternoon helps me feel better overall. Alleviates my stress and anxiety.” Some participants started new exercise routines, while others such as Janet (66y, F) strengthened existing practices: “I meditate every day and do yoga. But then I was trying to do that before. I'm much better at it now.” Some participants shifted from exercising in gyms to at home and outdoors, such as online classes and exercise apps. They described increased engagement in many forms of physically distant exercise including biking, fishing, playing golf, swimming, dancing, Pilates, running, weightlifting, and tai chi.

Participants strategically exercised alone or alongside a spouse, family members, or friends. Elizabeth (60y, F) described: “We have been hiking trails [Monday to Friday] with our dog and averaging 2–5 miles a hike. My husband tends to push me into going. I'm not in shape, so this has been hard on me, but in the end I'm ok with it.” Kathy (56y, F) shared: “[I'm] trying to drag the children for walks with me so I can be outside. Walking helps me cope. This is only marginally successful.”

Outdoor activities such as walking, hiking, yardwork, and biking were valued ways to be outside. Margaret (59y, F) shared: “I find getting outside in nature every day lifts my mood,” while Carolyn (56y, F) wrote: “getting outside of the house by walking in my yard, going for a motorcycle ride, and going for a car ride. All to make me feel like I am free from four walls and the television.” Participants such as Janice (66y, F) appreciated easy access to the outdoors: “I am in a rural area so I wander around outside, interact with wildlife/birds. Catch the sun coming up, wave it off as it goes down.” For Denise (73y, F): “Because the weather has been nice, I've been working in my garden more than usual. It gets me outside and gives me a feeling of accomplishment.” Being outside was described as therapeutic. David (63y, M) shared: “Being a photographer, [I] have been taking long walks in the woods with cameras, shooting wildlife, birds mostly! Takes my mind off of the state of the world right now and the huge loss of life.” Participants expressed finding reassurance and hope in the natural environment.

### Daily Life

Participants reported strategically restructuring and adapting their daily lives to cope with the pandemic. They described routine as a strategy to feel more normal or purposeful in everyday life. Many attempted to keep up regular work, sleep, and school schedules – even showering and dressing for work each day as usual. While work could help as a strategy to connect with others and maintain routine (particularly for those working remotely), it was not always stress-reducing: “I'm now working 12–14 h days, 7 days a week, so I am exhausted and often don't have time to think about the impact of the pandemic! Not a healthy coping device, but I'm terrified of losing my job” (Cynthia, 55y, F).

Other changes to daily life included “planning out fun activities through the day, [as] something to look forward to” (Sandra, 57y, F). Numerous participants made daily to-do lists and tried to keep busy to manage stress, stay occupied, and improve sleep. “I find if I do something productive everyday it helps me cope and feel better,” shared Pamela (62y, F). For Sharon (57y, F): “I am trying to stay on top of my feelings, I am trying to stay ahead of projects around the house, this keeps my mind busy and off of covid.” Sheltering in place was reframed as a chance by some to do projects not otherwise addressed: “I've been using the time to complete my ‘to do’ list, which is about 10 years old!” (Kathleen, 84y, F).

Reading and listening to audio content (e.g., music, podcasts, radio shows, and audiobooks) were daily coping strategies. They were pleasurable activities that could also provide temporary reprieve from the pandemic. Participants listened to soothing music, as well as meditation, prayer, and mindfulness programs. Cooking and baking were discussed: cooking more and different foods, trying new recipes, and trying to improve cooking skills. Carol (55y, F) shared her strategy of “cooking food with love and care” for family, while Diane (67y, F) reported “lots of food in fridge to cook from and be creative, cooking soups and cakes for neighbors.” Strategic and creative meals were a way to minimize grocery store shopping trips.

Additional strategic daily activities and hobbies included playing games, doing puzzles, watching television, painting, cleaning, knitting, crocheting, and sewing. Some participants described learning new hobbies, while others amplified existing hobbies such as woodworking, crafting, and playing instruments. Pets were valued companions to cope with the isolation: “Without my two dogs I would be going stir crazy. They are my support system and best companions ever” wrote Brenda (59y, F). Several participants had recently adopted dogs, and valued the comfort and busyness it added to their lives.

### COVID-19 Precautions

Reducing exposure to COVID-19 was a common coping strategy to protect oneself, family members, and others. Deborah's (74y, F) coping strategies: “Wear mask and gloves to grocery store. Washing hands constantly, using disinfecting wipes often.” Takeout, grocery delivery (by commercial service or family members), and avoiding crowds were methods to reduce risk. Participants described shopping during designated “senior hours” early in the morning. Robert (76y, M) shared “erecting firewalls between us and parcels handled by others. For instance, we bake our mail at 260F, oven off! For 15 minutes.” John (55y, M) described “cleaning like a mad man,” while others such as Barbara (67y, F) described disinfecting surfaces: “cleaning everything around the house and bleaching everything that comes into my home.” These activities helped to manage stress, limit fears of infection, and keep busy. But they also reflected uncertainty in the early stages of the pandemic and panic about how to stay safe. Some strategies, such as baking mail in the oven and bleaching all groceries (including vegetables and fruit) are not recommended public health strategies. Participants expressed concerns over misinformation and indecision about exactly which public health precautions to follow in the early months of the pandemic.

### Attitude and Outlook

Meditation, practicing mindfulness and patience, and breathing exercises were strategies to help manage stress and stay calm. Mary (58y, F) wrote that she is “listening to mindfulness podcasts, meditating more, [and] being honest with my family when I am feeling overwhelmed.” Participants described finding resources for daily meditation and mindfulness through podcasts, books, websites, and television shows. Some were new to meditation and learning, while others such as Linda (56y, F) expanded their existing routines:

I practice transcendental meditation twice a day; [it is] saving my life. All the stress is washed away. And after the meditation I am free of anxiety or stress from the day. Can't imagine not doing it during this time.

Relaxation techniques, affirmations of love and health, and focusing on gratitude helped some participants calm their thoughts when worry or panicking began. Patricia (74y, F) shared:

Along the lines of mindfulness, I remind myself how lucky I am to have shelter, food and friends; I remind myself to concentrate on only that which is in front of me, and which I can control; I remind myself often that this won't last forever.

Focusing on the present and ‘living in the moment’ were valued cognitive coping strategies. Michael (72y, M), for example, personally reframed social distancing as “an introvert's holiday.”

Susan (67y, F) tried to find humor in the situation: “occasionally venting to my sister using profuse swearing which makes my sister and eventually me laugh because I'm normally the nice person who seldom swears.” James (57y, M) shared his efforts: “trying to make people and myself laugh more – have some fun even if it's corny.” Humor was a way to individually feel better and connect with others.

### Social Connection

Staying connected to others virtually and in-person was a frequent coping strategy. Paul (68y, M) shared “[I'm] checking in with my family – it has been so hard to be away from them,” while Jane (84y, F) wrote “writing emails to friends with whom I usually connect only at Christmas.” Participants in this category discussed increased interactions through varied forms of communication including phone calls, texts, video chats (including weekly happy hours, game nights, and virtual babysitting), creating and sending videos, and handwritten letters. A few participants shared attending alcohol and drug rehabilitation support groups to maintain sobriety and get support: “I attend AA meeting via Zoom and I find it very helpful to share with others” (Fred, 61y, M). Social media and chat rooms were ways to connect with community members, friends/family, and strangers. Cathy (60y, F) shared:

I'm spending more time with two Facebook groups. One is a humor group and I laugh at a lot of the posts. The other is a Christian group with lots of reassuring scriptures and other encouraging posts. People post requests for prayer. Praying for them helps me feel more useful.

Online forums were ways to connect with others and find lightness and humor. “This will sound ridiculous, but every morning I do the [New York Times] crossword and Spelling Bee, then discuss the results via an online blog and with friends. It helps me start the day in an upbeat way” shared Robin (73y, F).

Interpersonal contact were strategies to connect with others, receive and provide emotional support and other forms of help, process and share news, and have fun. This included moving in with family members to help one another during quarantine, increased sexual activity, and activities such as pleasure car rides and games. “Playing at home games with my spouse at her request” wrote Larry (78y, M). Debbie (73y, F) shared: “[I] keep in touch with family and friends in various forms that are currently available, 3x weekly meet with neighbors maintaining social distancing to check in to see if anyone needs anything or knows of anyone who may be in need.” Participants such as Diana (57y, F) endeavored to visit and spend more time in-person with loved ones: “I visit my mom daily. We talk on the phone while [I] stand outside her window.” Outdoor walks were a way to see others while maintaining physical distance: “I try to walk with a friend (safely) about 3 times a week. The exercise and the bitch session are very helpful, but [I'm] still very depressed” expressed Marilyn (62y, F). For Kathryn (68y, F): “[I'm] setting up walks with a small group of friends at least twice weekly. Through a Women in Retirement group based out of a local senior center, I've made friends with like interests who are supporting each other during this pandemic.” Participants described deepening bonding and support with others as ways to cope with the pandemic.

### Online and Media Engagement

“I'm spending too much time on the computer” shared Anthony (87y, M). Participants described going online and using electronic devices to play games and puzzles, shop, find entertainment, and connect with others. Julie (63y, F) described her coping strategies as “joining online communities, learning to make sourdough starter and bread, joined master gardener online class on rain gardens and another on kitchen skills. Also reading my books I had on Kindle and watching some of the series on [television].” Watching shows and movies represented a way to improve mood and spend time with others. Gail (56y, F) described her coping as “A lot of TV! I know that's not a great idea, but it works for me.” Some participants watched live musical performances, holiday movies, comedies, and murder mysteries “to help keep our minds off of COVID-19” (William, 78y, M).

Others limited screen time as a coping strategy. This could help reduce distress about the pandemic and national politics. Joan (66y, F) shared: “I limit my time on social media and news sites because the volume of news is overwhelming.” For Richard (81y, M): “I don't listen to the news constantly. I believe the constant droning of news creates anxiety and depression. I check on things several times a day and focus on other things.” Participants shared finding reliable sources of information as a coping strategy to stay informed about COVID-19 and government responses to the pandemic.

### Faith-Based Practices

Spiritually-minded participants shared coping through faith-based practices. “I am watching more Christian programming and listening to worship music with scripture while I go to sleep” said Judy (68y, F). Rebecca (62y, F) shared that she was “praying for all family, friends, and everyone in the world.” Participants prayed more, and for a breadth of people ranging from family members and friends to healthcare professionals, first responders, those at risk (including older adults), and those sick with COVID-19. Joyce (66y, F) shared: “I do a lot of praying more than I[‘m] use[d] to doing. I thank God that all of my kids are fine. I lost a sister last week because of the COVID-19 and they can't bury her because so many people are dying in New York.” Prayer was a way to cope with grief and feel hope for the future. Participants also found solace in attending virtual religious gatherings and study sessions. Most participants in this category expressed following Christian practices, while others adhered to Buddhist practices.

### Projects and Learning

Home projects and learning activities were described as easy coping strategies. Participants shared studying new languages and musical instruments, taking online courses, and working on hobbies (e.g., poetry, writing, drawing, skateboarding, gardening). For Steven (60y, M), coping included “learn[ing] computer skills to better work from home and connecting with others.” Learning something new and striving for improvement helped some keep both mind and body busy, and able to enjoy a sense of accomplishment, progress, or control.

“I am trying to stay on top of my feelings, I am trying to stay ahead of projects around the house. This keeps my mind busy and off of covid” wrote Cindy (57y, F). Decluttering, cleaning, organizing, spring cleaning, painting and general home projects were methods to keep busy in a safe manner, focus on the present, feel a sense of accomplishment, and generate improvements. Martha (62y, F) shared: “Getting projects started and completed around the house and yard that I don't usually get a chance to do. Enjoy getting the feeling of being caught up for once.” For Bonnie (66y, F): “For me activity helps – yard work and home improvement projects have been instrumental in my sanity.” Gary (71y, M) was sorting and cleaning his late wife's studio: “It's been good as I needed to tackle all of this, I wasn't ready to until now. Feels very good but I also have the great sadness of missing her. Not ready to join her though!!”

### Civic Engagement

Participants such as Karen (76y, F) sought coping strategies to help others and feel purposeful: “I organized through our neighborhood social network site a group to make cloth masks to cover N95s for medical professionals, first responders, police, etc., and have been making those masks as well.” Nancy (58y, F) also made masks: “I am disabled so I am limited to what I can do. I've just been trying to keep busy by sewing masks for family and friends and the neighborhood children.” Donna (64y, F) shared: “I'm making masks. I have no control over the virus or [the President], but can exercise some control by making masks to help others – and myself.” Participants shared that making masks felt important and meaningful.

Others described donating money and goods, supporting local businesses, and volunteering. Respondents checked in with family, friends, and colleagues to offer support and reassurance; baked and cooked for others; and shared local resources in their communities. Some participants described coping through political engagement: contacting elected officials, volunteering on political campaigns, and attending protests.

### Food, Alcohol, and Substances

Healthy eating, antioxidants, vitamins, nutritional supplements, essential oils, and staying hydrated were described as coping strategies to nourish one's body, stay active, maintain routine, and boost the immune system. Teresa (58y, F) engaged in “self-care through proper eating, hydrating, and physical movement to stay healthy and build immunity.” Christine (74y, F) shared “taking additional supplements to enhance my immune system.” Ordering takeout meals was a method to limit in-person grocery store contact, enjoy new and different foods, and support local businesses: “[I'm] ordering take-out and delivery to help local eateries and to treat myself, started eating super power smoothies to keep healthy” (Catherine, 70y, F).

Some participants tried to avoid overeating with varying levels of success. Several mentioned accomplishments of healthier food habits, while others such as Shirley (65y, F) lamented “I'm eating a lot of chocolate (sigh).” Food was an emotional comfort for participants such as Judith (67y, F): “Eating more alleviates anxiety when alone.” Some participants described increased alcohol consumption (e.g., beer, wine, and cocktails) to relax, enjoy an evening routine, or “get drunk.” For Betty (57y, F), “I make dinner and clean up, pour a glass of wine and watch a movie (or 2) then go to bed.” Lisa (66y, F) described coping through cannabis: “daily happy hour smoking weed sometimes as early as 3 pm start, usually 4–5 pm until midnight. Helps sleep and watching [the news] without totally freaking out.” Marijuana use was described as a method to elevate mood, calm anxiety, feel better, and relax.

### Health and Wellness

Physical and mental health were important priorities. In addition to exercise, meditative practices, healthy eating, and taking COVID-19 precautions (described in previous categories), participants shared specific health practices. Some participants described trying to sleep more, nap, boost sleep quality, and maintain a sleep schedule. This was perceived as important to rest and recovery, a way to improve overall health, boost immune function, and pass the time. Theresa (59y, F) shared: “Doc prescribed a sleep aid which I take infrequently. Sleep got better once I found a way to help” through making and donating masks. She expressed that “seeing SO many people struggling with the issues I struggle with every day (isolation, boredom, anxiety, depression) has been oddly validating.” Participants such as Connie (57y, F) sought increased help from health professionals and support groups: “returned to weekly, from bi-monthly, therapy sessions.” Anne (61y, F) “reached out to crisis helpline and community mental help for support with depression and recovery from extended illness due to undiagnosed COVID-19.” Participants described seeing therapists, acupuncture specialists, and chronic disease specialists. Gloria (64y, F) “had to call [a] doctor to be put on anxiety meds.” Others described taking Cannabidiol (CBD) supplements, sleep aids, and anti-anxiety medications to help relax and sleep.

### Planning

Making future travel and long-term plans, in addition to estate planning and contingency plans, were coping strategies in response to the pandemic. Thomas (68y, M) shared: “[I'm] keeping busy planning for when things will be normal. This includes vacation planning.” Strategic thinking about the future included planning for shifted retirement income and locations, post-lockdown events, road trips, and different scenarios to accommodate the uncertainty ahead. “Focusing on the long-term beyond the current pandemic and economic crisis” helped Mark (71y, M) to cope. Participants such as Paula (82y, F) shared reviewing and writing estate plans, wills, and medical care directives: “Updated my will and advance directive (for peace of mind).” For Peggy (72y, F): “[I'm] making sure that my family know what and where my end of life documents and wishes.” Participants such as Charles (79y, M) coped by planning for worst-case scenarios: “Made a contingency plan if I get Covid-19 to protect my wife.” Coping strategies included making plans for sickness and sharing wishes for care preferences with family members if they became seriously ill from COVID-19.

### Nothing

Over twenty percent of participants reported *not* using any coping strategies. Some participants expressed feeling fine given their attitudes and personalities: “I'm a pretty relaxed, easy going person by nature. I'm normally into healthy living and the past few months haven't been any different” wrote Daniel (78y, M). Participants such as Timothy (74y, M) described previous self-isolation tendencies and introverted lifestyle preferences:

I am mostly an introvert, with a superficially extroverted facade to deal with social situations. So, this period of enforced time without external “invasions” of my time is welcome. On the other hand, it sure is a crappy way to have achieved it. In other words, my coping is mostly doing what I usually do, and enjoying it.

Some participants who already lived alone expressed comfort and longstanding practice with isolation: “Since I have lived alone for years, I am comfortable with this. While I do enjoy going out regularly, I miss it, but my attitude is this isn't forever and it's what is needed for my health” (Dorothy, 75y, F). For George (59y, M): “I am disabled, and so I am at home as usual. No[t] much change.” This echoed Wanda (63y, F), who expressed: “There has been NO lifestyle change in my life as what everyone else is now doing is what I did every day otherwise. I am disabled.” Jean (55y, F) who managed a long-term chronic illness felt that life was very similar: “In fact, now that so much is online, the world has opened up for me in many ways.”

Some participants expressed not needing coping strategies because they were not afraid of coronavirus, felt it was overblown, or not much of a threat. Some reported not having any issues or needs to address. Others drew upon lifetime experiences: “I don't have any issues coping with COVID-19, it's just another bump in the road of life, I'm 74 years old and have been through much worse” wrote Kevin (74y, M). For Douglas (89y, M):

I am a person who experienced the Great Depression and WWII, and I am a student of history. When I was growing up, the 1918 flu epidemic was often a topic of conversation. My life experiences have conditioned me to deal with this pandemic without panic.

Finally, some participants of this category did not have strategies because they were not sure where to start or how to cope. They expressed feeling helpless, lost, that nothing was working, or unable to afford any strategies. “Don't know what to do. No money” wrote Sheila (68y, F). Virginia (59y, F) responded: “None. My mother passed away in April and the sadness is overwhelming since we cannot be together with family.” Others felt frustrated when unable to participate in their established coping strategies such as volunteering, team and gym athletics, and social groups.

## Discussion

### Coping Among Older Adults

While older adults are more physically vulnerable to COVID-19, this study contributes to an emerging counternarrative to the often-bleak portrayal of older adults (4). We highlight diverse and wide-ranging sources of strength and resilience among older adults to cope with adverse psychosocial, sociocultural, behavioral, and socioeconomic consequences of the pandemic. Consistent with the transactional model of stress and coping (28), participants employed both cognitive and behavioral strategies. Active coping behaviors included getting exercise and going outside, adjusting daily routines, taking public health precautionary measures, and fostering social connections. Participants also shared cognitive strategies such as reframing their attitude and outlook.

These results are consistent with Aldwin's (29) five main categories: problem-focused coping (behaviors and cognitions targeted toward solving or managing a problem, such as implementing a plan), emotion-focused coping (managing one's emotional reaction to the problem), social support coping (eliciting others' help or providing support to others), religious coping (seeking help from a higher power, such as praying), and cognitive reframing (trying to make sense of the problem and/or focus on the situation's positive aspects). These strategies are not mutually exclusive, and participants often reported using more than one concurrently or sequentially.

Our findings validate and extend understandings of coping and psychological resilience among aging populations. Resilience is a multifaceted and important concept: high resilience in later life has been associated with reduced risk for depression and mortality, better self-perceptions of aging successfully, increased quality of life, and improved lifestyle behaviors (8, 11, 13). Previous research suggests that older adults are more resilient than younger adults, including higher emotional regulation and problem-solving approaches to cope with adversity (11). Our study participants adapted strategies to unique pandemic circumstances, such as volunteering to make masks and socializing through video calls instead of in-person activities. The majority of participants expressed adaptive coping skills, which deepens the counternarrative of older adults as strong and resilient (as opposed to vulnerable, frail, weak, and disposable) in the COVID-19 pandemic.

### Coping Challenges Since the Pandemic Onset

Older adults vary in the extent to which they are able to access and use coping strategies amid the pandemic. While some participant coping involved no immediate participation costs (e.g., walking outside, practicing mindfulness, cleaning), others were less accessible, such as having home ownership and the financial means to undergo home renovation projects, private outdoor space to spend time in (e.g., backyards and gardens), or workplace support and technological capacity to work from home. Further, even “no-cost” strategies may be less accessible to socioeconomically marginalized populations, such as living close to green space and in neighborhoods with safe walkable streets to be physically active and access resources (30, 31). Forms of outdoor recreation can often require expensive equipment and access to a vehicle. Heightened unequal access to resources during the pandemic may exacerbate pre-existing disparities to cope with adversity and build psychological resilience among older populations (12).

Further, not all coping strategies were health-promoting. Previous research suggests that those who endorse more wishful thinking, avoidance, denial, and substance abuse are associated with worse health (32). Some participants described relying on health-limiting strategies, or both functional and dysfunctional coping simultaneously. Evidence-based interventions designed to build and strengthen positive coping strategies and resilience among older adults are severely lacking (8). Previous methods, such as in-person senior center programs, may need to be adapted and tailored given fundamental changes to communities, daily life, and aging since the pandemic onset. Given the popularity and perceived benefits of nature connection and outdoor experiences among our participants (and the still-pressing need for physical distancing in communities across the globe given lack of widespread vaccination), ecotherapy techniques (33) may have particular utility during this time to promote coping and resilience among older adults. We need greater awareness, investigation, and discussion of coping since the pandemic onset among researchers, clinicians, community health providers, and most importantly, older adults themselves.

### Strengths and Limitations

The study fills a knowledge gap on resilience during COVID-19 from the perspectives of aging adults (8, 11). The qualitative content analysis generated knowledge based upon participants' unique perspectives (16). Rather than using a deductive approach to test validated scales such as the Brief Coping Orientation to Problems Experienced (Brief COPE) inventory (34) or Ways of Coping Scale (35), we employed an inductive approach to develop a framework that captures the complexity and diversity of participants' emotional and behavioral reactions during the first upswing of the COVID-19 pandemic in the US. Many of our participant-generated categories overlapped the Brief COPE domains including self-distraction, active coping, denial, substance use, use of emotional support, venting, planning, humor, and religion. One notable divergence from validated scales is the immense scale of the stressor (i.e., a global pandemic) and that participants could not directly control or alleviate the problem (i.e., take action to eliminate the pandemic). This could elicit feelings of lacking agency, frustration, fear, and helplessness. However, many participants focused on employing secondary strategies to control what they could (e.g., being safe in their own home, keeping busy, adjusting their outlook) that contributed to feelings of coping efficacy (36).

The study has important limitations. We launched this study during the first upswing of the pandemic and did not capture people who may have been too sick to participate, such as those who were hospitalized with COVID-19 or other health conditions. Men, older adults from racial and ethnic minority groups, Spanish speakers, and those with high school education or less were under-represented relative to the general population. The strategies used to cope with the COVID-19 pandemic that we observed in this study may be employed at different frequencies among these groups in the general population, and we may not have observed relevant coping strategies among these population groups. Under-representation of the perspectives of key population groups who have been identified as being more vulnerable to physical disease and associated socioeconomic harms of the pandemic may limit consideration of important perspectives and bias our results toward the perspectives of more advantaged older adults. Results of this study should be triangulated against those from other study populations and sociodemographic groups.

The sample size is extremely large in comparison to traditional qualitative studies, and our results derived from a single open-ended question. This limited deep, in-depth, case-oriented analysis in the current study (37). Response richness was further limited by the online survey format because we could not probe participants for further inquiry and follow-up. We did not write up the “Other” category in the results given the lack of a consistent pattern in this grouping except for 19 participants who stated that they did not understand the question. Many of the categories overlapped each other. We endeavored to group themes and participant ideas commonly expressed together into categories, but the boundaries imposed by the coding structure may artificially separate the interrelatedness of coping mechanisms. The efficacy of self-reported coping strategies remains unknown, as well as distinction in coping strategies expressed by different groups (e.g., young-old vs. old-old, working vs. not employed, frail vs. well). Further research should build upon this exploratory analysis to focus on variables associated with particular coping strategies (e.g., age, gender, race, ethnicity, income, marital status, geographic location) and health outcomes (e.g., anxiety, depression, self-rated health).

Strengths of this study include its timeliness: data collection occurred early in the pandemic when coping strategies may have been especially critical to deal with immense social, economic, political, and public health upheaval. Our general inductive approach (22) was responsive to potentially-novel coping strategies employed since the pandemic onset, and enabled nuance in the findings (such as concurrent coping strategies). The wide age range of participants accounts for a breadth of aging experiences and perspectives, such as those who are working and retired, caring and being cared for, and those with high- to limited-mobility. The national coverage and large sample size enhance the generalizability of our findings.

## Conclusions and Implications

The sources of resilience and coping strategies in this study have potential practical implications to promote well-being and quality of life among aging adults during the pandemic and future societal traumas. Older adults may benefit from interventions that harness positive coping strategies such as walking outdoors, adjusted daily routines, breathing exercises, and staying socially connected (8, 11). Communities can support coping by creating the social infrastructure for mutual support and transmission reduction. For example, grocery stores have created special shopping hours for older and at-risk populations, while some communities have organized mutual-aid groups to deliver groceries, medications, and other supplies to vulnerable populations (38). Policymakers could strengthen infrastructure to connect vulnerable populations to essential resources and services, deliver clear public health messaging, invest in more equitable neighborhood infrastructure to encourage regular physical activity, and provide more programming to promote social cohesion. Our results suggest that these coping mechanisms helped foster meaningful activities, social support, and decreased anxiety and worry. Public health, educational, and counseling programs might incorporate these coping strategies to support mental health and well-being among older adults. Programs might provide tools to develop and nurture daily routine, sleep, diet, exercise, social connection, meaningful activity, and self-care skills during and after the pandemic.

Poor mental health impacts of COVID-19 may extend for years beyond the pandemic, as evidenced following previous crises including severe acute respiratory syndrome and the 9/11/2001 attacks on the US (5, 6). Health professionals and policymakers need to be informed about specific and diverse types of coping strategies employed by older adults in response to potentially long-term adverse impacts of the pandemic. This knowledge is critical to bolster coping strategies that promote connectedness, self-reliance, and purpose, while at the same time incorporating consideration of established personal preferences, autonomy, and capabilities of older adults. Including the perspectives of older adults themselves in planning and delivery of mental health services strengthens such efforts.

## Data Availability Statement

The raw data supporting the conclusions of this article will be made available by the authors, without undue reservation.

## Ethics Statement

The studies involving human participants were reviewed and approved by University of Michigan Health Sciences and Behavioral Sciences Institutional Review Board. The patients/participants provided their written informed consent to participate in this study.

## Author Contributions

LK and JF conceived of, designed, and supervised the COVID-19 Coping Study. JF, JK, BO'S, ME, YV, and LK conducted the analysis. JF drafted the manuscript with input from JK, BO'S, and LK. All authors contributed to the interpretation of data, revision of the manuscript for important intellectual content, and have read and approved of the final version of the manuscript.

## Conflict of Interest

The authors declare that the research was conducted in the absence of any commercial or financial relationships that could be construed as a potential conflict of interest.

## Footnotes

^1^Bracketed information following participant pseudonyms denotes age and sex reported at the time of survey completion.
